# GPT-4 vs. radiologists: who advances mediastinal tumor classification better across report quality levels? a cohort study

**DOI:** 10.1097/JS9.0000000000003127

**Published:** 2025-08-08

**Authors:** Ru Wen, Xiaoming Li, Kang Chen, Manhong Sun, Chunxia Zhu, Peng Xu, Fengxi Chen, Can Ji, Mi Pei, Xuefeng Li, Xiaojuan Deng, Quan Yang, Weixiang Song, Yajun Shang, Sheng Huang, Mingyang Zhou, Jian Wang, Chaoyang Zhou, Wei Chen, Chen Liu

**Affiliations:** a 7T Magnetic Resonance Translational Medicine Research Center, Department of Radiology, Southwest Hospital, Army Medical University (Third Military Medical University), Chongqing, China; b College of Mathematics and Statistics, Chongqing University, Chongqing, China; c Department of Radiology, the Third Affiliated Hospital of Chongqing Medical University, Chongqing, China; d Department of Radiology, The Affiliated Yongchuan Hospital of Chongqing Medical University, Chongqing, China; e Department of Radiology, Second Affiliated Hospital of Chongqing Medical University, Chongqing, China; f Department of Radiology, The People’s Hospital of Tong liang District, Chongqing, China; g School of Big Data and Software Engineering, Chongqing University, Chongqing, China; h Department of Medicine, Yidu Cloud (Beijing) Technology Co. Ltd., Beijing, China

**Keywords:** diagnostic accuracy, GPT-4, mediastinal tumors, radiological reports, tumor classification

## Abstract

**Background:**

Accurate mediastinal tumor classification is crucial for treatment planning, but diagnostic performance varies with radiologists’ experience and report quality.

**Purpose:**

To evaluate generative pretrained transformer’s (GPT-4’s) diagnostic accuracy in classifying mediastinal tumors from radiological reports compared to radiologists of different experience levels using radiological reports of varying quality.

**Materials and methods:**

We conducted a retrospective study of 1494 patients from five tertiary hospitals with mediastinal tumors diagnosed via chest CT and pathology. Radiological reports were categorized into low-, medium-, and high-quality based on predefined criteria assessed by experienced radiologists. Six radiologists (two residents, two attending radiologists, and two associate senior radiologists) and GPT-4 evaluated the chest CT reports. Diagnostic performance was analyzed overall, by report quality, and by tumor type using Wald *χ*^2^ tests and 95% CIs calculated via the Wilson method.

**Results:**

GPT-4 achieved an overall diagnostic accuracy of 73.3% (95% CI: 71.0–75.5), comparable to associate senior radiologists (74.3%, 95% CI: 72.0–76.5; *P* >0.05). For low-quality reports, GPT-4 outperformed associate senior radiologists (60.8% vs. 51.1%, *P*<0.001). In high-quality reports, GPT-4 was comparable to attending radiologists (80.6% vs.79.4%, *P*>0.05). Diagnostic performance varied by tumor type: GPT-4 was comparable to radiology residents for neurogenic tumors (44.9% vs. 50.3%, *P*>0.05), similar to associate senior radiologists for teratomas (68.1% vs. 65.9%, *P*>0.05), and superior in diagnosing lymphoma (75.4% vs. 60.4%, *P*<0.001).

**Conclusion:**

GPT-4 demonstrated interpretation accuracy comparable to Associate Senior Radiologists, excelling in low-quality reports and outperforming them in diagnosing lymphoma. These findings underscore GPT-4’s potential to enhance diagnostic performance in challenging diagnostic scenarios.

**Summary:**

In this retrospective study involving 1494 chest CT reports of different quality from five tertiary hospitals, GPT-4 demonstrated diagnostic accuracy comparable to Associate Senior Radiologists in classifying mediastinal tumors from chest CT reports, excelling in low-quality reports and outperforming Associate Senior Radiologists in diagnosing specific tumor types like lymphoma, showcasing its potential to enhance diagnostic performance in challenging scenarios.

## Introduction

Mediastinal tumors present unique challenges in imaging diagnosis due to their distinctive anatomical structure, complex tissue composition, and low incidence^[1–3]^. Mediastinal tumors originate from diverse tissues and exhibit a variety of tissue features, therefore, integrating the precise localization with morphological imaging features is crucial for the differentiation and classification of these tumors. The proper classification of these tumors can then provide essential guidance in appropriate treatment strategies^[4–6]^. The diagnostic accuracy of radiologists varies significantly across experience levels, and the complexity of mediastinal tumors likely exacerbates these discrepancies, posing a challenge for ensuring accurate diagnoses. Developing simple and effective methods to improve diagnostic accuracy for mediastinal tumors is critical, both for advancing research and improving patient outcomes^[7,8]^.

Moreover, the incidence of primary mediastinal tumors, including thymoma, lymphoma, neurogenic tumor, and teratoma is relatively low compared to tumors such as lung and breast cancer, which can pose significant challenges for artificial intelligence (AI) models in the diagnosis of primary mediastinal tumors^[9–11]^. Recent advancements in AI, particularly those in general natural language processing models such as generative pretrained transformer-4 (GPT-4), present significant opportunities to enhance the accuracy and efficiency of radiological diagnoses^[12,13]^. By utilizing large-scale data training, GPT-4 has the potential to mitigate gaps in clinical experience and can assist in complex diagnostic tasks that traditionally rely on the specialized expertise of radiologists. Research has demonstrated that large language models (LLMs) can accurately generate automated synoptic reports[14]. However, one recent comparison suggested that GPT-4 and radiologists achieved similar diagnoses of chest X-rays in some cases[15]. LLMs also have the potential to detect errors within radiology reports as well. GPT-4 has performed exceptionally well in identifying errors in radiology reports, which could substantially enhance the accuracy of these reports[16]. Furthermore, LLMs have shown strong potential in mining free-text data, and GPT-4 has excelled in mining free-text data from lung cancer computed tomography (CT) reports. These data suggest that LLMs have potentially broad applications in data mining and information extraction[17]. The low incidence of mediastinal tumors results in a lack of sufficient diagnostic experience and knowledge accumulation among radiologists in routine clinical practice[18], warranting a need for further information to help diagnose with more accuracy. We hypothesize that the GPT model with rich knowledge can effectively assist physicians in improving the accuracy of mediastinal tumor diagnosis, if successfully implemented, such models may not only enhance diagnostic performance but also provide educational value through interactive learning^[19,20]^, allowing clinicians to compare its output with their own evaluations and pathological results-thereby improving diagnostic reasoning over time and offering real-world clinical benefits, particularly by supporting junior physicians and facilitating the integration of AI into daily workflows[21].

To assess this, the current study utilizes histopathological diagnosis as the gold standard to evaluate the diagnostic accuracy of GPT-4 in detecting mediastinal tumors. By comparing GPT-4’s performance with radiologists of varying levels of expertise, we aim to determine whether GPT-4 can match or surpass the diagnostic capabilities of experienced radiologists. With this research, we hope to aid in the consistency and accuracy of radiological diagnoses in clinical settings. To ensure transparency and reproducibility, this study adheres to the TITAN 2025 guideline for the reporting of artificial intelligence research in medicine[22].

**HIGHLIGHTS**

- We conducted a retrospective study involving 1494 patients with mediastinal tumors from five tertiary hospitals, where GPT-4 and six radiologists with varying experience levels analyzed chest CT-based radiological reports of differing quality, validated against pathological diagnoses.
- GPT-4 demonstrated overall diagnostic accuracy comparable to Associate Senior Radiologists (73.3% vs. 74.3%, *P >* 0.05), outperforming them in low-quality reports (60.8% vs. 51.1%, *P <* 0.001) and comparable to attending radiologists in high-quality reports (80.6% vs. 79.4%, *P >* 0.05).
- GPT-4 outperformed Associate Senior Radiologists in diagnosing lymphoma (75.4% vs. 60.4%, *P < 0.001*) while showing comparable accuracy to radiology residents for neurogenic tumors (44.9% vs. 50.3%, *P > 0.05*) and to Associate Senior Radiologists for teratomas (68.1% vs. 65.9%, *P >* 0.05).

## Materials and methods

### Study population

We conducted a retrospective analysis of 1494 patients with mediastinal tumors from five tertiary hospitals (Hospital A, Hospital B, Hospital C, Hospital D, Hospital E). All patients underwent chest CT imaging between January 2012 and December 2023. The study population consisted of patients diagnosed with one of the following four primary mediastinal tumor subtypes: thymoma, lymphoma, neurogenic tumors, and teratomas. The ground truth diagnosis for each case was established based on histopathological confirmation, either through surgical resection or image-guided biopsy, which was reviewed and verified by board-certified pathologists at the respective institutions. Inclusion criteria included high-resolution chest CT images and corresponding radiological reports. Exclusion criteria included patients who had undergone tumor resection surgery prior to the study. This multi-classification study of primary mediastinal tumor was conducted in accordance with the principles of the Helsinki Declaration and approved for data use by the local ethics committee (Ethics Committee of the first affiliated hospital of Military Medical University [BKY 2024298]). This work has been reported in line with the STARD (Standards for the Reporting of Diagnostic accuracy studies) criteria. This study conforms to the TITAN 2025 Guidelines.

### Radiological report categorization

**Figure 1.**
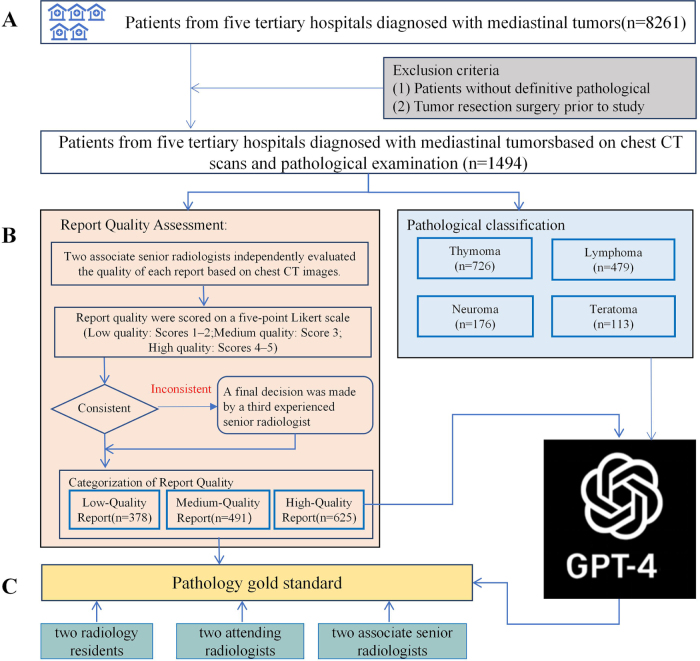
Flowchart of study design. (A) Initially, 1494 original radiology reports of patients with mediastinal tumors, all of which had been pathologically diagnosed with specific types of mediastinal tumors, were included. (B) Chest CT images were independently reviewed by two experienced thoracic radiologists to assess the quality of the original radiology reports. The reports were categorized into three quality levels: low, medium, and high. Based on the pathological gold standard, the mediastinal tumors were classified as thymoma, lymphoma, neurogenic tumor, or teratoma. (C) GPT-4 and three radiologists were tasked with determining the specific type of mediastinal tumor for each radiology report.

Before the quality of patients’ chest CT imaging reports was assessed, two associate senior radiologists, Dr. 1 (with 15 years of diagnostic experience and over 75 000 chest imaging assessments) and Dr. 2 (with 14 years of diagnostic experience and over 50 000 chest imaging assessments), independently evaluated the quality of each chest CT report. During the evaluation process, Dr. 1 and Dr. 2 meticulously reviewed the corresponding chest CT images and examined the written report content. Based on predefined criteria, the reports were categorized into three quality levels: low, medium, and high. The predefined criteria included clarity of description, completeness of imaging findings, and the level of detail in lesion characterization, specifically encompassing morphology, size, precise location within the mediastinum, boundaries, relationships with adjacent structures, and degree of enhancement. By simultaneously reviewing the chest CT images and the corresponding report content, Dr. 1 and Dr. 2 were able to more comprehensively assess the clarity of report descriptions, the completeness of imaging findings, and the level of detail in lesion characterization, thereby ensuring the thoroughness and accuracy of the report quality assessments. Report quality was assessed using a five-point Likert scale (with 1 indicating the lowest quality and 5 the highest quality). Based on the Likert scale scores, reports were classified into three categories: low quality (scores 1–2), medium quality (score 3), and high quality (scores 4–5). In cases of disagreement between the initial evaluators, a final decision was made by a third experienced senior radiologist, Dr. 3 (with 39 years of diagnostic experience). Additionally, six radiologists, including two associate senior radiologists (Dr. 4 with 15 years of radiological diagnostic experience, Dr. 5 with 15 years of radiological diagnostic experience), two attending radiologists (Dr. 6 with 9 years of radiological diagnostic experience, Dr. 7 with 10 years of radiological diagnostic experience), and two radiology residents (Dr. 8 with 3 years of radiological diagnostic experience, Dr. 9 with 2 years of radiological diagnostic experience) and GPT-4 were tasked with diagnosing mediastinal tumors through chest CT reports. Diagnostic performance overall, across different report quality levels, and for specific mediastinal tumor types was assessed. Figure 1 presents the flowchart of the experimental design.

### CT scanning protocol

Chest enhanced-CT examinations were conducted using multiple CT systems from different vendors and countries of origin. All scans utilized a tube voltage of 120 kVp and employed automatic tube current modulation. The reconstruction slice thickness was 1–1.5 mm, with reconstruction interval set at 1–1.25 mm. Pitch values ranged from 1.0 to 1.55. Intravenous contrast agents used included iodinated compounds with iodine concentrations of 320–370 mg/mL, administered at an injection rate of 3–4.5 mL/s with a total volume of 65–95 mL. Refer to the Supplemental Digital Content Table 1, available at: http://links.lww.com/JS9/E850 for detailed parameters.

### Model configuration and prompt design

**Figure 2.**
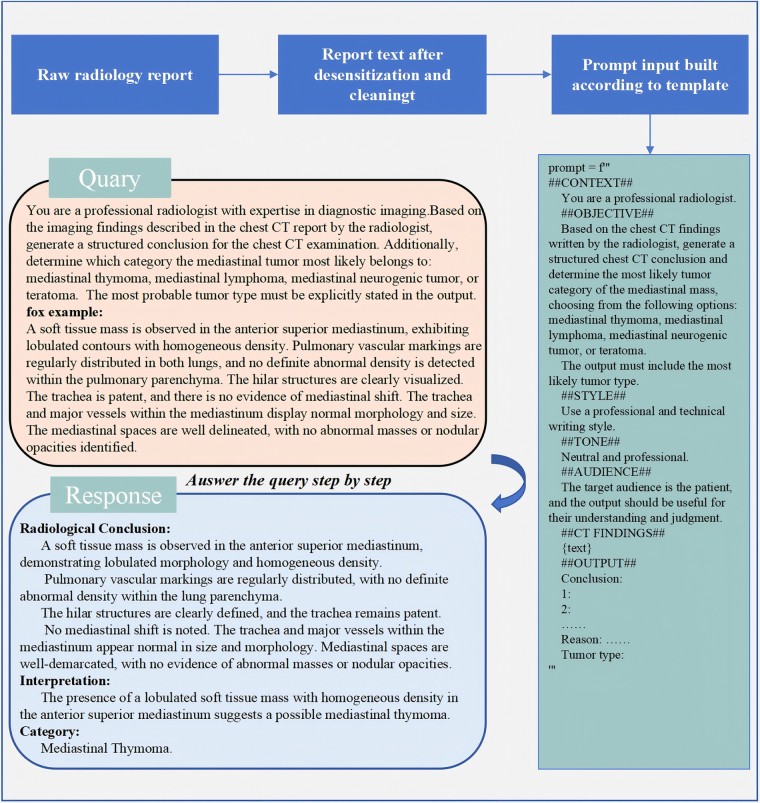
Illustrates the complete data processing pipeline, beginning with the original radiology reports (containing identifiable information), followed by de-identification, text cleaning, prompt formatting, and culminating in the structured conclusions and classification output generated by GPT-4.

To ensure the standardization of data input and the interpretability of the model’s output, we designed a comprehensive data preprocessing and prompt construction pipeline, as illustrated in Figure 2. Additional technical details are provided in the supplemental Digital Content Material 2, available at: http://links.lww.com/JS9/E851.

We implemented a two-step prompting framework with GPT-4, accessed via the OpenAI API (model identifier: GPT-4). First, the model was instructed to convert the free-text CT findings into a structured diagnostic conclusion. Then, based solely on this structured output, GPT-4 was prompted to classify the mediastinal tumor into one of four categories: mediastinal thymoma, lymphoma, neurogenic tumor, or teratoma. This approach ensures the classification is grounded in interpretable, radiologically structured information.

All prompts followed a standardized template that defined the model’s context, task objective, style, tone, and output format (Supplemental Digital Content Table 2, available at: http://links.lww.com/JS9/E850). GPT-4 was invoked with the following parameters: *temperature* = 0.1, *max_tokens* = 1024, *top_P* = 0.9, and *frequency_penalty* = 0. These settings were chosen to promote deterministic outputs and minimize linguistic variability across repeated generations. No fine-tuning or model adaptation was performed. To ensure consistency and reproducibility, a structured prompt engineering strategy was employed. Prompts followed a fixed template that included a brief instruction, followed by the corresponding free-text radiology report. All prompt executions were conducted in a controlled environment using automated scripts to reduce bias and eliminate variability due to manual input. The detailed code can be found in Supplemental Digital Content Material 3, available at: http://links.lww.com/JS9/E852. The use of a low temperature and fixed model parameters ensured stable and repeatable outputs across the dataset. This approach allowed us to evaluate GPT-4’s diagnostic reasoning under standardized conditions, enhancing the methodological rigor and transparency of the study.

### Statistical analysis

All analyses were conducted using IBM SPSS Statistics version 26.0 (IBM) and Python version 3.9 (Python). Our study involved nine radiologists, including those responsible for report quality assessment and diagnostic evaluation. The report evaluators consisted of two associate senior radiologists and an experienced senior radiologist. The six diagnostic radiologists included two radiology residents, two attending radiologists, and two associate senior radiologists. To assess the consistency between the two associate senior radiologists (Dr. 1 and Dr. 2) who evaluated the quality of radiological reports, we calculated the interobserver agreement using the Intraclass Correlation Coefficient (ICC). We assessed the mediastinal tumor diagnostic performance of radiologists within each subset of experience levels. Our outcome measures included the number of correctly diagnosed mediastinal tumors and diagnostic accuracy rate. Each participant’s diagnostic performance was compared with that of GPT-4 in the context of mediastinal tumor diagnosis. We used the Wald *χ*^2^ test to compare the diagnostic performance of GPT-4 and radiologists. Specifically, GPT-4 diagnostic outcomes were compared with radiologist outcomes at each experience level. To evaluate the accuracy of the diagnostic results, we calculated 95% confidence intervals (CIs) using the Wilson method. We conducted our analysis across all report quality levels as well as independently for low-, medium-, and high-quality reports. Additionally, we performed subgroup analyses on mediastinal tumor subtypes (thymoma, lymphoma, neurogenic tumor, and teratoma). We separated mediastinal tumors into subgroups to further assess the diagnostic accuracy of GPT-4 compared to radiologists with varying levels of diagnostic experience. Three LLMs – GPT-4, GPT-3.5, and GPT-o1 – were evaluated. GPT-4 was the primary focus, and its diagnostic performance was compared against that of radiologists as well as GPT-3.5 and GPT-o1. This study systematically evaluated the classification performance of GPT-4 in interpreting imaging reports of mediastinal tumors using several commonly adopted metrics, including accuracy, precision, recall, and F1 score. To further assess the model’s diagnostic capability, receiver operating characteristic (ROC) curves were constructed and the area under the curve (AUC) was calculated. Statistical comparisons of AUC values between GPT-4 and radiologists with varying levels of experience were conducted using DeLong’s test to ensure analytical rigor and comparability. In addition, to explore the potential clinical utility of GPT-4 in diagnostic decision support, the Net Reclassification Improvement (NRI) metric was introduced. NRI was employed to evaluate GPT-4’s performance in correctly reclassifying cases relative to human readers, detailed methods are provided in the Supplementary Methods (Supplemental Digital Content Material 1, available at: http://links.lww.com/JS9/E850).

## Results

### Overall diagnostic performance

Overall, GPT-4 demonstrated a diagnostic accuracy rate of 73.3% (1095 of 1494; 95% CI: 71.0–75.5) for the detection of mediastinal tumors. This was comparable to the average Associate Senior Radiologist (74.3% [1110.5 of 1494; 95% CI: 72.0–76.5], *P* > 0.05). However, the accuracy of GPT-4 in diagnosing tumors was significantly higher than in Radiology Residents (59.2% [884.0 of 1494; 95% CI: 56.7–61.7], *P* < 0.0001) and Attending Radiologists (64.5% [963.5 of 1494; 95% CI: 62.0–66.9], *P* < 0.0001).

### Diagnostic performance by report quality

The resulting ICC of 0.734 (95% CI: 0.686–0.773, *P* < 0.001) reflects good agreement between the two independent thoracic radiologists, suggesting that the categorization into low-, medium-, and high-quality levels was consistent and reproducible. Table 1 illustrates the diagnostic performance of radiologists and GPT-4 under varying conditions of report quality. GPT-4 outperformed radiologists at all experience levels on low-quality reports, achieving a diagnostic accuracy of 60.8% (230 of 378, 95% CI: 55.8–65.6). Its accuracy was significantly higher than the diagnostic accuracy of Radiology Residents (22.0%, 83 of 378, 95% CI: 18.1–26.4, *P* < 0.0001) and Attending Radiologists (35.7%, 135 of 378, 95% CI: 31.0–40.7, *P* < 0.0001); but slightly lower than Associate Senior Radiologists (51.1%, 193 of 378, 95% CI: 46.1–56.1, *P* < 0.001); For medium-quality reports, GPT-4 demonstrated a diagnostic accuracy of 73.5% (361 of 491, 95% CI: 69.4–77.2). This rate was comparable to Associate Senior Radiologists (76.2%, 374 of 491, 95% CI: 72.2–79.8, *P* > 0.05). However, GPT-4 significantly outperformed Radiology Residents (66.3%, 325.5 of 491, 95% CI: 62.0–70.3, *P* < 0.001) and Attending Radiologists (67.6%, 332.0 of 491, 95% CI: 63.3–71.6, *P* < 0.01) for medium-quality reports; In high-quality reports, GPT-4 performed with an accuracy rate of 80.6% (504 of 625, 95% CI: 77.3–83.5). This rate was lower than that of Associate Senior Radiologists (87.0%, 543.5 of 625, 95% CI: 84.1–89.4, *P* < 0.0001). However, GPT-4’s performance was similar to Attending Radiologists (79.4%, 496.5 of 625, 95% CI: 76.1–82.4, *P* > 0.05) for high-quality reports.

**Table 1:** Comparison of diagnostic accuracy rates between radiologists and GPT-4 across different report quality levels

Reader	Total (*n* = 1494)		Low-quality report (*n* = 378)		Medium-quality report (*n* = 491)		High-quality report (*n* = 625)	
	Diagnostic accuracy rate (%)	*P* value*	Diagnostic accuracy rate (%)	*P* value	Diagnostic accuracy rate (%)	*P* value	Diagnostic accuracy rate (%)	*P* value
Resident 1	59.2 (56.7,61.7) [885/1494]	<0.0001	22.0 (18.1,26.4) [83/378]	<0.0001	66.6 (62.3,70.6) [327/491]	<0.01	76.0 (72.5,79.2) [475/625]	<0.01
Resident 2	59.1(56.6,61.6) [883/1494]	<0.0001	22.0 (18.1,26.4) [83/378]	<0.0001	66.0(61.7,70.1) [324/491]	<0.001	76.2 (72.7,79.4) [476/625]	<0.05
Radiology Resident average	59.2 (56.7,61.7) [884.0/1494]	<0.0001	22.0 (18.1,26.4) [83.0/378]	<0.0001	66.3 (62.0,70.3) [325.5/491]	<0.001	76.1 (72.6,79.3) [475.5/625]	<0.05
Attending 1	64.1 (61.6,66.5) [957/1494]	<0.0001	39.9 (35.1,44.9) [151/378]	<0.0001	65.8 (61.5,69.9) [323/491]	<0.001	77.3 (73.9,80.4) [483/625]	>0.05
Attending 2	64.9 (62.4,67.3) [970/1494]	<0.0001	31.5 (27.0,36.3) [119/378]	<0.0001	69.5 (65.3,73.4) [341/491]	<0.05	81.6 (78.4,84.4) [510/625]	>0.05
Attending radiologist average	64.5 (62.0,66.9) [963.5/1494]	<0.0001	35.7 (31.0,40.7) [135.0/378]	<0.0001	67.6 (63.3,71.6) [332.0/491]	<0.01	79.4 (76.1,82.4) [496.5/625]	>0.05
Senior 1	75.0 (72.7,77.1) [1121/1494]	>0.05	52.1 (47.1,57.1) [197/378]	<0.001	76.4 (72.4,79.9) [375/491]	>0.05	87.8 (85.0,90.1) [549/625]	<0.0001
Senior 2	73.6 (71.3,75.8) [1100/1494]	>0.05	50.0 (45.0,55.0) [189/378]	<0.001	76.0 (72.0,79.6) [373/491]	>0.05	86.1 (83.2,88.6) [538/625]	<0.0001
Associate senior radiologist average	74.3 (72.0,76.5) [1110.5/1494]	>0.05	51.1 (46.1,56.1) [193.0/378]	<0.001	76.2 (72.2,79.8) [374.0/491]	>0.05	87.0 (84.1,89.4) [543.5/625]	<0.0001
GPT-4	73.3 (71.0,75.5) [1095/1494]		60.8 (55.8,65.6) [230/378]		73.5 (69.4,77.2) [361/491]		80.6 (77.3,83.5) [504/625]	

Data in parentheses represent 95% confidence intervals (CIs), while those in brackets indicate numerators and denominators. *P* values were adjusted for multiple comparisons using the Bonferroni correction.

* We used the Wald *χ*^2^ test to compare the diagnostic accuracy of GPT-4 and the radiologists in identifying mediastinal tumor categories.

### Diagnostic performance by tumor type

**Figure 3.**
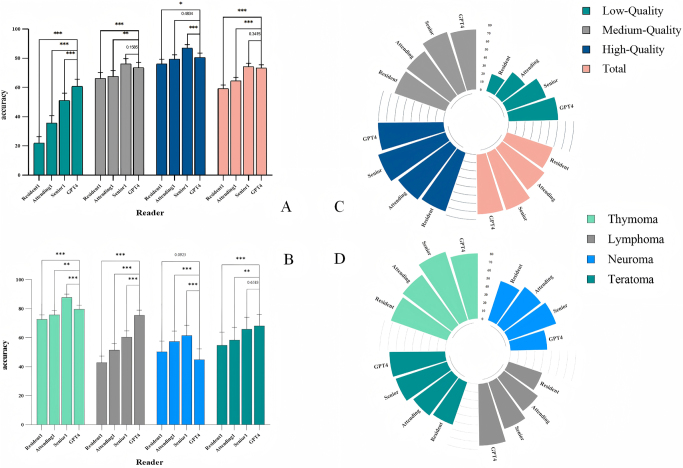
(A) The bar chart illustrates the diagnostic accuracy of GPT-4 and radiologists across reports of varying quality. (B) The bar chart illustrates the diagnostic accuracy of GPT-4 and radiologists across different types of mediastinal tumors. (C) A comparison of diagnostic accuracy between radiologists and GPT-4 across different levels of report quality using circular bar plots. (D) A comparison of classification accuracy for mediastinal tumors between radiologists of varying experience levels and GPT-4 using circular bar plots. *** indicates *P* < 0.001, ** indicates *P* < 0.01, and * indicates *P* < 0.05.

**Figure 4.**
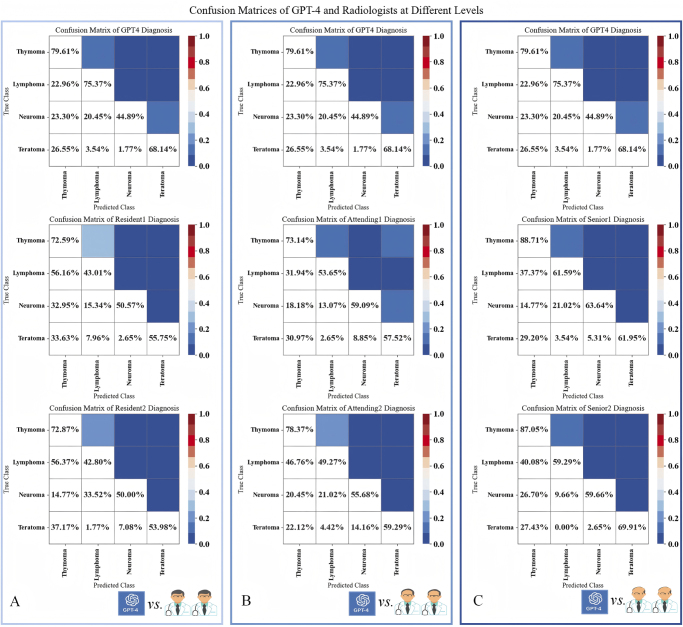
Confusion matrices showing the classification performance of GPT-4 and each radiologist across mediastinal tumor types. Rows represent ground truth labels, and columns represent predicted labels. The matrices highlight misclassification patterns and diagnostic consistency across groups.A: GPT-4 and Resident 1, Resident 2; B: GPT-4 and Attending 1, Attending 2; C: GPT-4 and Senior 1, Senior 1.

Figure 3 illustrates the diagnostic performance of GPT-4 and radiologists across different report qualities, tumor types, and experience levels. The classification performance of GPT-4 in diagnosing various pathological subtypes of mediastinal tumors is presented in Supplemental Digital Content Table 3, available at: http://links.lww.com/JS9/E850 and Supplemental Digital Content Figure 1, available at: http://links.lww.com/JS9/E850. GPT-4 achieved a diagnostic accuracy of 79.6% (578 of 726, 95% CI: 76.5–82.4) for thymoma, slightly lower than the average rate at Associate Senior Radiologists (87.9%, 638 of 726, 95% CI: 85.3–90.1; *P* < 0.001), however, GPT-4 outperformed the average diagnostic accuracy of Radiology Residents (72.7%, 528 of 726, 95% CI: 69.3–75.8; *P* < 0.001). Interestingly, GPT-4’s accuracy was similar to that of Attending Radiologists (75.8%, 550 of 726, 95% CI: 72.6–78.8; *P* < 0.05); GPT-4 demonstrated the highest diagnostic accuracy for assessment of lymphoma, with an average accuracy of 75.4% (361 of 479, 95% CI: 71.4–79.0). This average was significantly better than the average seen for Associate Senior Radiologists (60.4%, 289.5 of 479, 95% CI: 56.0–64.7; *P* < 0.001), Radiology Residents (42.9%, 205.5 of 479, 95% CI: 38.5–47.4; *P* < 0.001), and Attending Radiologists (51.5%, 246.5 of 479, 95% CI: 47.0–55.9; *P* < 0.001); GPT-4 achieved a diagnostic accuracy of 44.9% (79 of 176, 95% CI: 37.7–52.3) for neurogenic tumors. This rate was significantly lower than the average diagnostic accuracy for Associate Senior Radiologist (61.6%, 108.5 of 176, 95% CI: 54.2–68.5; *P* < 0.001) and Attending Radiologists (57.4%, 101.0 of 176, 95% CI: 50.0–64.5; *P* < 0.001). However, GPT-4’s performance was similar to the accuracy of Radiology Residents (50.3%, 88.5 of 176, 95% CI: 43.0–57.6; *P* > 0.05); GPT-4 achieved a diagnostic accuracy of 68.1% for assessment of teratomas (77 of 113, 95% CI: 59.0–76.0), which was similar to the average diagnostic accuracy of Associate Senior Radiologists (65.9%, 74.5 of 113, 95% CI: 56.8–74.0; *P* > 0.05). However, GPT-4 significantly outperformed both Radiology Residents (54.9%, 62 of 113, 95% CI: 45.7–63.8; *P* < 0.001) and Attending Radiologists (58.4%, 66 of 113, 95% CI: 49.2–67.1; *P* < 0.01). Table 2 provides a comprehensive summary of the diagnostic performance of GPT-4 compared to Associate Senior Radiologists, Radiology Residents, and Attending Radiologists across different histological subtypes. Figure 4 presents the confusion matrices for GPT-4 and each radiologist across all tumor types. These matrices visualize the distribution of true and misclassified predictions, enabling direct comparison of diagnostic patterns. DeLong’s test results (Supplemental Digital Content Table 4, available at: http://links.lww.com/JS9/E850) demonstrated that GPT-4 achieved significantly superior diagnostic performance compared to residents and attending radiologists in most tumor categories, while showing comparable accuracy to associate senior radiologists, particularly in the diagnosis of thymoma and teratoma (all *P* > 0.05).

**Table 2:** Comparison of diagnostic accuracy rates between radiologists of varying experience levels and GPT-4 for mediastinal tumor categories

Reader	Thymoma (*n* = 726)		Lymphoma (*n* = 479)		Neuroma (*n* = 176)		Teratoma (*n* = 113)	
	Diagnostic accuracy rate (%)	*P* value^*^	Diagnostic accuracy rate (%)	*P* value	Diagnostic accuracy rate (%)	*P* value	Diagnostic accuracy rate (%)	*P* value
Resident 1	72.6 (69.2,75.7) [527/726]	<0.001	43.0 (38.6,47.5) [206/479]	<0.001	50.6 (43.3,57.9) [89/176]	>0.05	55.8 (46.6,64.6) [63/113]	<0.01
Resident 2	72.9 (69.6,76.0) [529/726]	<0.001	42.8 (38.4,47.3) [205/479]	<0.001	50.0 (42.7,57.3) [88/176]	>0.05	54.0 (44.8,62.9) [61/113]	<0.001
Radiology Resident average	72.7 (69.3,75.8) [528.0/726]	<0.001	42.9 (38.5,47.4) [205.5/479]	<0.001	50.3 (43.0,57.6) [88.5/176]	>0.05	54.9 (45.7,63.8) [62.0/113]	<0.001
Attending 1	73.1 (69.8,76.2) [531/726]	<0.001	53.7 (49.2,58.1) [257/479]	<0.001	59.1 (51.7,66.1) [104/176]	<0.001	57.5 (48.3,66.2) [65/113]	<0.05
Attending 2	78.4 (75.3,81.2) [569/726]	>0.05	49.3 (44.8,53.8) [236/479]	<0.001	55.7 (48.3,62.8) [98/176]	<0.001	59.3 (50.1,67.9) [67/113]	<0.01
Attending Radiologist average	75.8 (72.6,78.8) [550.0/726]	<0.05	51.5 (47.0,55.9) [246.5/479]	<0.001	57.4 (50.0,64.5) [101.0/176]	<0.001	58.4 (49.2,67.1) [66.0/113]	<0.01
Senior 1	88.7 (86.2,90.8) [644/726]	<0.001	61.6 (57.2,65.8) [295/479]	<0.001	63.6 (56.3,70.3) [112/176]	<0.001	61.9 (52.7,70.3) [70/113]	>0.05
Senior 2	87.1 (84.5,89.3) [632/726]	<0.001	59.3 (54.8,63.6) [284/479]	<0.001	59.7 (52.3,66.7) [105/176]	<0.001	69.9 (60.9,77.6) [79/113]	>0.05
Associate Senior Radiologist average	87.9 (85.3,90.1) [638.0/726]	<0.001	60.4 (56.0,64.7) [289.5/479]	<0.001	61.6 (54.2,68.5) [108.5/176]	<0.001	65.9 (56.8,74.0) [74.5/113]	>0.05
GPT-4	79.6 (76.5,82.4) [578/726]		75.4 (71.4,79.0) [361/479]		44.9 (37.7,52.3) [79/176]		68.1 (59.0,76.0) [77/113]	

### Diagnostic performance of benign and malignant tumors

GPT-4 achieved a diagnostic accuracy of 86.3% for benign mediastinal tumors (876 of 1015, 95% CI: 84.0–88.3), which was comparable to the average performance of Attending Radiologists (85.5%, 868 of 1015, 95% CI: 83.2–87.5; *P* > 0.05), and lower than that of Associate Senior Radiologists (90.8%, 922.0 of 1015, 95% CI: 88.9–92.4; *P* < 0.001). Notably, GPT-4 significantly outperformed Radiology Residents, whose average diagnostic accuracy was 77.6% (788.0 of 1015, 95% CI: 74.9–80.1; *P* < 0.001). In contrast, for malignant mediastinal tumors, GPT-4 demonstrated a markedly higher diagnostic accuracy of 75.4% (361 of 479, 95% CI: 71.4–79.0), outperforming all human readers across experience levels. Associate Senior Radiologists achieved an average accuracy of 60.4% (289.5 of 479, 95% CI: 56.0–64.7; *P* < 0.001), followed by Attending Radiologists at 51.5% (246.5 of 479, 95% CI: 47.0–55.9; *P* < 0.001), and Radiology Residents at 42.9% (205.5 of 479, 95% CI: 38.5–47.4; *P* < 0.001). Among individual readers, the highest-performing human reader (Senior 1) reached 61.6% (295 of 479, 95% CI: 57.2–65.8), still significantly lower than GPT-4. Table 3 provided a comprehensive summary of the diagnostic accuracy rates of GPT-4 and radiologists with varying levels of experience in differentiating benign and malignant mediastinal tumors. Supplemental Digital Content Table 5, available at: http://links.lww.com/JS9/E850 provided the diagnostic performance metrics between radiologists of different experience levels and GPT-4 in differentiating benign and malignant mediastinal tumors.

**Table 3:** Comparison of diagnostic accuracy rates between radiologists of different experience levels and GPT-4 in differentiating benign and malignant mediastinal tumors

Reader	benign tumor (*n* = 1015)		Malignant tumor (*n* = 479)	
	Diagnostic accuracy rate (%)	*P* value	Diagnostic accuracy rate (%)	*P* value
Resident 1	78.4(75.8,80.8) [796/1015]	<0.001	43.0(38.6,47.5) [206/479]	<0.001
Resident 2	76.8(74.1,79.3) [780/1015]	<0.001	42.8(38.4,47.3) [205/479]	<0.001
Radiology resident average	77.6(74.9,80.1) [788.0/1015]	<0.001	42.9(38.5,47.4) [205.5/479]	<0.001
Attending 1	88.1(86.0,89.9) [894/1015]	>0.05	53.7(49.2,58.1) [257/479]	<0.001
Attending 2	83.0(80.6,85.2) [842/1015]	<0.05	49.3(44.8,53.8) [236/479]	<0.001
Attending radiologist average	85.5(83.2,87.5) [868.0/1015]	>0.05	51.5(47.0,55.9) [246.5/479]	<0.001
Senior 1	89.7(87.7,91.4) [910/1015]	<0.05	61.6(57.2,65.8) [295/479]	<0.001
Senior 2	92.0(90.2,93.5) [934/1015]	<0.001	59.3(54.8,63.6) [284/479]	<0.001
Associate senior radiologist average	90.8(88.9,92.4) [922.0/1015]	<0.001	60.4(56.0,64.7) [289.5/479]	<0.001
GPT-4	86.3(84.0,88.3) [876/1015]		75.4(71.4,79.0) [361/479]	

### Diagnostic Performance of GPT-4 Versus GPT-3.5 and GPT-o1

Overall, GPT-4 achieved a diagnostic accuracy of 73.3% (1095/1494; 95% CI: 71.0–75.5) for mediastinal tumor classification, significantly outperforming GPT-3.5 (45.8%; 95% CI: 43.3–48.3; *P* < 0.0001) and GPT-o1 (65.0%; 95% CI: 62.5–67.4; *P* < 0.0001).

Stratified by report quality, GPT-4 consistently demonstrated superior diagnostic accuracy compared to GPT-3.5 and GPT-o1 (Supplemental Digital Content Table 7, available at: http://links.lww.com/JS9/E850). In low-quality reports, GPT-4 achieved an accuracy of 60.8% (230/378; 95% CI: 55.8–65.6), significantly higher than GPT-3.5 (47.6%; 95% CI: 42.6–52.6; *P* < 0.0001) and GPT-o1 (55.8%; 95% CI: 50.8–60.7; *P* = 0.0133). In medium-quality reports, GPT-4 reached 73.5% accuracy (361/491; 95% CI: 69.4–77.2), again outperforming GPT-3.5 (43.0%; 95% CI: 38.7–47.4; *P* < 0.0001) and GPT-o1 (67.0%; 95% CI: 62.7–71.0; *P* < 0.01). For high-quality reports, GPT-4 achieved its highest accuracy of 80.6% (504/625; 95% CI: 77.3–83.5), significantly exceeding GPT-3.5 (46.9%; 95% CI: 43.0–50.8; *P* < 0.001) and GPT-o1 (69.0%; 95% CI: 65.3–72.5; *P* < 0.001).

The diagnostic accuracy of GPT-4 was further evaluated across specific mediastinal tumor categories. For thymoma (*n* = 726), GPT-4 achieved an accuracy of 79.6% (578/726; 95% CI: 76.5–82.4), significantly higher than GPT-3.5 (38.2%; 95% CI: 34.7–41.8; *P* < 0.001) and GPT-o1 (53.7%; 95% CI: 50.1–57.3; *P* < 0.001). In lymphoma (*n* = 479), GPT-4 achieved 75.4% accuracy (361/479; 95% CI: 71.4–79.0), significantly lower than GPT-o1 (78.9%; 95% CI: 75.0–82.3; *P*< 0.05) but higher than GPT-3.5 (68.1%; 95% CI: 63.8–72.1; *P* < 0.001). For neuroma (*n* = 176), GPT-4 reached 44.9% accuracy (79/176; 95% CI: 37.7–52.3), outperforming GPT-3.5 (29.0%; 95% CI: 22.8–36.1; *P* < 0.001) but lower than GPT-o1 (60.8%; 95% CI: 53.4–67.7; *P* < 0.001). In teratoma (*n* = 113), GPT-4 achieved 68.1% accuracy (77/113; 95% CI: 59.0–76.0), significantly higher than GPT-3.5 (26.5%; 95% CI: 19.2–35.3; *P* < 0.001) but lower than GPT-o1 (85.0%; 95% CI: 77.3–90.4; *P* < 0.001) (Supplemental Digital Content Table 8, available at: http://links.lww.com/JS9/E850).

## Discussion

In this study, we evaluated GPT-4’s performance in interpreting imaging reports of mediastinal tumors and compared it to radiologists with varying levels of experience. GPT-4 achieved an overall accuracy in interpreting imaging reports comparable to that of Associate Senior Radiologists (73.3% vs. 74.3%, *P* > 0.05) and outperformed both radiology residents and attending radiologists. As shown in Supplemental Digital Content Table 6, available at: http://links.lww.com/JS9/E850, GPT-4 demonstrated positive NRI values compared to: Radiology residents (NRI + 0.14); Attending radiologists (NRI + 0.09) However, GPT-4 showed minimal or negative NRI relative to associate senior radiologists (NRI−0.01), suggesting comparable performance with experienced clinicians but substantial improvement over junior readers. These trends paralleled accuracy improvements and highlight GPT-4’s utility in enhancing diagnostic quality in less experienced settings.

Beyond comparisons with human readers, we also evaluated the diagnostic accuracy of three LLMs – GPT-4, GPT-o1, and GPT-3.5 – in classifying mediastinal tumors based on radiology reports of varying quality. GPT-4 demonstrated superior or competitive diagnostic performance across all tumor categories compared with GPT-3.5 and GPT-o1. These findings highlight GPT-4’s potential as a robust clinical decision support tool, especially when provided with high-quality textual input[23]. The superior performance of GPT-4 may be attributed to its architectural and training advancements. Although the exact specifications of GPT-4 remain undisclosed, previous studies have shown that GPT-4 is likely to have a significantly larger parameter size, longer context window, and enhanced instruction-following capability compared to its predecessors[24]. These upgrades contribute to improved comprehension of nuanced clinical narratives and greater diagnostic accuracy, particularly when synthesizing multi-sentence, unstructured radiology text. GPT-3.5, by contrast, was significantly less accurate across all report quality levels and tumor categories. This is consistent with prior evaluations showing limited performance of earlier language models in domain-specific medical tasks due to insufficient training in clinical syntax and semantic disambiguation[25]. Interestingly, GPT-o1 showed relatively strong performance in certain tumor subtypes, especially teratomas, where it outperformed GPT-4 (85.0% vs. 68.1%). This suggests that GPT-o1 may have undergone additional domain-specific fine-tuning, making it more sensitive to characteristic patterns in specific tumor descriptions. However, its overall generalization capacity was inferior to GPT-4, indicating a potential trade-off between specialization and broad diagnostic capability.

Notably, GPT-4 demonstrated exceptional performance in analyzing low-quality reports (*P* < 0.001) and showed higher accuracy in identifying clues related to lymphomas and teratomas, suggesting its potential utility in interpreting certain pathologies. These findings highlight GPT-4’s promise as an adjunct tool for supporting radiologists, particularly in scenarios where radiologist’s experience is limited or report complexity is high. A particularly noteworthy observation is that GPT-4 outperformed radiologists at all experience levels on low-quality reports. GPT-4 achieved an accuracy of 60.8% in identifying textual clues from these reports, which was markedly higher than that of associate senior radiologists (51.1%), attending radiologists (35.7%), and radiology residents (22%). These findings suggest that GPT-4 demonstrates robustness in interpreting imaging reports with incomplete or poorly articulated information, which is a common challenge in clinical practice. GPT-4’s robust performance could be in part due to its advanced deep learning algorithms, which are better equipped to extract and synthesize relevant textual clues from complex and ambiguous information compared to radiologists’ interpretation of such reports[26]. Previous studies have evaluated the performance of GPT-4 in extracting clinical phenotypes from electronic health records, demonstrating its effectiveness in analyzing textual data in contexts with limited or incomplete information. These findings underscore GPT-4’s robustness in synthesizing clues from complex or ambiguous reports[27]. Findings such as this are consistent with earlier research highlighting the high sensitivity and accuracy of AI algorithms in interpreting imaging reports and identifying unreported or mislabeled findings in chest imaging. The use of AI in such cases can help reduce errors arising from missed or misinterpreted information by radiologists. This result likely reflects AI’s ability to leverage its extensive knowledge base and advanced pattern recognition techniques to extract and synthesize relevant textual clues, thereby compensating for human physicians’ limitations in analyzing similar reports[26]. For imaging reports of moderate quality, GPT-4’s accuracy in interpreting the reports was comparable to that of Associate Senior Radiologists (73.5% vs. 76.2%, *P* > 0.05); however, GPT-4’s performance still exceeded that of radiology residents (50.3%, *P* < 0.001) and attending radiologists (57.4%, *P* < 0.001). For high-quality reports, GPT-4’s interpretation performance was equivalent to that of attending radiologists (80.6% vs. 79.4%, *P* > 0.05), highlighting that while AI is a powerful tool, human expertise and experience can still play important roles in particular cases, particularly when all relevant clinical details are well-documented^[28,29]^. Nonetheless, GPT-4 outperformed resident and attending radiologists in interpreting imaging reports, suggesting that in cases with sufficient and clear information, human experience plays a critical role in report interpretation, but GPT-4 consistently performs at an above-average level. Further studies comparing ChatGPT with radiologists in neuroradiology cases have shown that while GPT-4’s accuracy in interpreting imaging reports is lower than that of experienced radiologists, the difference is not statistically significant. In particularly challenging cases, GPT-4’s ability to extract and analyze relevant textual clues from imaging reports has not yet matched that of Associate Senior Radiologists[30].

Moreover, GPT-4 exhibits variable performance in interpreting imaging reports for different tumor types. For thymomas, GPT-4 achieved an accuracy of 79.6%, which was slightly lower than that of associate senior radiologists (87.9%) but still outperformed residents and attending radiologists. These data suggest that while GPT-4 is effective in extracting and synthesizing textual clues, experienced radiologists may be better at recognizing nuanced information specific to thymomas. These results also align with research emphasizing the importance of experience in the nuanced interpretation of certain tumor types. The high accuracy observed for both human radiologists and GPT-4 in interpreting thymomas may be attributed to the distinctive textual descriptions associated with thymomas in imaging reports. Thymomas are often described as well-defined anterior mediastinal masses with homogeneous enhancement, making them relatively easier to identify in report text[31]. Because of these specific imaging characteristics described in the reports, both radiologists and AI algorithms can often accurately interpret them. However, the marginally higher accuracy of human experts underscores the importance of clinical experience and potentially explains the higher accuracy observed among Associate Senior Radiologists. These specialists may be better able to integrate subtle textual clues from imaging reports with clinical context – an ability that AI, while advanced, may not fully replicate at this time. In contrast, GPT-4 demonstrated superior performance in interpreting imaging reports related to lymphoma, achieving an accuracy of 75.4%, which is significantly higher than that of associate senior radiologists (60.4%) and markedly better than radiology residents (42.9%) and attending radiologists (51.5%).

These findings indicate that GPT-4 may be particularly adept at extracting and synthesizing textual clues from imaging reports that suggest the likelihood of lymphoma, even when such patterns are less apparent to human observers. This could be attributed to the ability of AI to process and analyze complex textual data within large datasets, providing a supplementary advantage in report interpretation for such cases[32]. For teratoma diagnosis, GPT-4 reached an accuracy of 68.1%, comparable to that of associate senior radiologists (65.9%). However, in neurogenic tumors, GPT-4’s accuracy dropped to 44.9%, equivalent to a resident radiologist (50.3%), which was notably lower than associate senior radiologists (61.6%) and attending radiologists (57.4%). GPT-4’s lower accuracy in classifying neurogenic tumors may be attributed to several factors. Although neurogenic tumors are typically located in the posterior mediastinum and often described in imaging reports as well-defined, homogeneous masses[33], they still lack consistent and distinctive descriptive language compared to thymoma or lymphoma, these relatively nonspecific imaging features may create difficulty for AI algorithms, such as GPT-4, to interpret imaging reports and distinguish neurogenic tumors from other posterior mediastinal tumors. In addition, the relatively smaller number of neurogenic tumor cases in our dataset (*n* = 176) may have resulted in weaker representation learning. Lastly, experienced radiologists can leverage their expertise to identify subtle anatomical and morphological clues embedded in the report content, resulting in higher accuracy when assessing the likelihood of tumor types^[34,35]^ – such as tumor location and bone changes – with prior experience, whereas GPT-4, without access to structured clinical data, is currently unable to replicate this level of diagnostic reasoning. The lower accuracy of GPT-4 observed in this category highlights the current limitations of AI in interpreting imaging reports. While AI serves as a powerful adjunct tool, it cannot yet replicate the nuanced judgment and clinical reasoning of experienced radiologists when analyzing complex or ambiguous cases based on report content^[36,37]^. These limitations may primarily due to the model’s limited ability to generalize across diverse and nuanced clinical scenarios described in imaging reports. Therefore, future research should focus on optimizing the training datasets for AI models to enhance their ability to extract and interpret textual clues from imaging reports, particularly in complex and ambiguous cases.

Building on this potential, a key consideration is how GPT-4 could be practically integrated into clinical workflows to maximize its utility as a diagnostic aid. While GPT-4 demonstrated diagnostic accuracy comparable to experienced radiologists, particularly in low-quality reports and for specific tumor types such as lymphoma, it is not intended to replace human experts. Instead, its optimal role may lie in augmenting clinical decision-making, especially when radiologist experience is limited or report quality is suboptimal. One potential integration strategy involves deploying GPT-4 as a triage or decision-support tool within the radiology workflow. For instance, GPT-4 could provide preliminary classification suggestions, highlight ambiguous or atypical cases for further review, or offer differential diagnoses to assist junior radiologists. Such a collaborative framework would allow radiologists to retain full clinical oversight while benefiting from AI-driven consistency and speed. Future research may further explore how AI-human synergy can be optimized across different clinical scenarios and tumor categories[38]. In summary, the findings of this study underscore the potential of GPT-4 as an adjunct tool in radiology, particularly in scenarios where human expertise may be constrained by experience or the quality of available imaging reports. While radiologists may not routinely assess other radiologists’ reports to determine the likelihood of specific mediastinal tumor types.

### Learning points

- GPT-4 demonstrates diagnostic performance comparable to associate senior radiologists in classifying mediastinal tumors from CT reports.
- In low-quality report scenarios, GPT-4 surpasses even associate senior radiologists, showcasing its resilience to suboptimal documentation.
- The model shows particular strength in identifying lymphoma, where it outperforms all human comparator groups.
- These findings suggest GPT-4 can serve as a valuable adjunct tool to radiologists, especially in resource-limited or high-variance reporting environments.
- Integration of AI like GPT-4 may enhance consistency and accuracy in complex diagnostic workflows, paving the way for hybrid decision-making models.

## Conclusion

GPT-4’s strong performance in interpreting low-quality imaging reports and identifying textual clues indicative of particular tumor types, such as lymphomas, suggests broader applicability in other clinical contexts. The model’s performance suggests potential utility in clinical scenarios where imaging reports are suboptimal or where access to experienced radiologists is limited. This tool and others may lead to improved patient outcomes; however, the variability observed in performance across different tumor types and report qualities highlights the need for ongoing refinement and validation of AI models in clinical practice. GPT-4 holds great promise as a valuable assistant to radiologists, improving the accuracy and efficiency of imaging report interpretation. Our study indicates that while GPT-4 is a promising tool, it should complement, rather than replace, human expertise in radiologic workflows. Further research into seamlessly integrating GPT-4 into clinical practice will be crucial for maximizing its utility in supporting radiologists.

## Data Availability

The datasets during the current study are available from the corresponding author upon reasonable request.

## Research registration unique identifying number (UIN)

Not applicable. This study is a retrospective analysis, which involves the examination of existing data collected for purposes other than the current research. According to the World Medical Association’s Declaration of Helsinki 2013, Article 35, the requirement for registration in a publicly accessible database applies primarily to prospective studies that involve the recruitment of human subjects. Since our study does not involve prospective recruitment or intervention, but rather analyzes previously collected data, it is not suitable for clinical trial registration. We have ensured that all ethical considerations and approvals were obtained for the original data collection, and the study adheres to all relevant ethical guidelines and regulations. Therefore, the requirement for a Unique Identifying Number (UIN) from a clinical trial registry is not applicable to this retrospective study.
